# CB_1_ Receptor Autoradiographic Characterization of the Individual Differences in Approach and Avoidance Motivation

**DOI:** 10.1371/journal.pone.0042111

**Published:** 2012-07-27

**Authors:** Daniela Laricchiuta, Maria Luisa Rojo, Antonio Rodriguez-Gaztelumendi, Fabio Ferlazzo, Laura Petrosini, Christopher J. Fowler

**Affiliations:** 1 Centro Europeo per la Ricerca sul Cervello (CERC)/Santa Lucia Foundation, Rome, Italy; 2 Department of Psychology, University “Sapienza” of Rome, Rome, Italy; 3 Department of Pharmacology and Clinical Neuroscience, Umeå University, Umeå, Sweden; Kaohsiung Chang Gung Memorial Hospital, Taiwan

## Abstract

Typically, approach behaviour is displayed in the context of moving towards a desired goal, while avoidance behaviour is displayed in the context of moving away from threatening or novel stimuli. In the current research, we detected three sub-populations of C57BL/6J mice that spontaneously responded with avoiding, balancing or approaching behaviours in the presence of the same conflicting stimuli. While the balancing animals reacted with balanced responses between approach and avoidance, the avoiding or approaching animals exhibited inhibitory or advance responses towards one of the conflicting inputs, respectively. Individual differences in approach and avoidance motivation might be modulated by the normal variance in the level of functioning of different systems, such as endocannabinoid system (ECS). The present research was aimed at analysing the ECS involvement on approach and avoidance behavioural processes. To this aim, in the three selected sub-populations of mice that exhibited avoiding or balancing or approaching responses in an approach/avoidance Y-maze we analysed density and functionality of CB_1_ receptors as well as enzyme fatty acid amide hydrolase activity in different brain regions, including the networks functionally responsible for emotional and motivational control. The main finding of the present study demonstrates that in both approaching and avoiding animals higher CB_1_ receptor density in the amygdaloidal centro-medial nuclei and in the hypothalamic ventro-medial nucleus was found when compared with the CB_1_ receptor density exhibited by the balancing animals. The characterization of the individual differences to respond in a motivationally based manner is relevant to clarify how the individual differences in ECS activity are associated with differences in motivational and affective functioning.

## Introduction

The superordinate division of emotions is distributed along a bipolar dimension of affective valence, from approaching to avoiding stimuli. Approaching can be defined as the tendency to direct behaviour towards rewarding situations, objects and possibilities, whereas avoiding as the tendency to direct behaviour away from fearful situations, objects and possibilities. It is postulated that approach and avoidance behaviours determine one's disposition to the primary emotions of fear, anger and attachment and one's behavioural responses to the environmental stimuli related to danger, novelty and reward [1]. The likelihood of a subject in engaging in approach or avoidance behaviours varies across situations, individuals and lifespan. Notably, individual differences may be important predictors of vulnerability to neuropsychiatric disorders, including depression, anxiety and addiction, especially upon exposure to environmental adversity [2].

It is hypothesized that approach or avoidance behaviours are associated with the brain networks that control cognitive and attentional functions, reward sensitivity and emotional expression and involve cortico-limbic circuitry including prefrontal cortex, amygdala and striatum [3], [4]. There is considerable variability among individuals in the cortico-limbic system reactivity to novel and emotional stimuli, although the origin of the individual differences is not yet fully clarified.

Growing evidence indicates that the endocannabinoid system (ECS) plays an important role in the control of balancing between approach and avoidance both in humans [5], [6] and in rodents [7]–[10], as well as in the control of emotional processes such as anxiety, extinction of aversive memories and stress coping [11]–[15]. Furthermore, ECS is involved in tuning behaviours mediated by reward central networks [16]–[18], in particular in the rewarding properties of palatable foods [19], [20], as well as in physiological functions related to neuro-protection and neuro-inflammation [21], [22]. After their biosynthesis from arachidonic acid, endocannabinoids, such as anandamide (AEA) and 2-arachidonoylglycerol (2-AG), typically modulate synaptic neurotransmission through stimulation of cannabinoid type-1 receptors (CB_1_) [17], [18], [23]–[26], densely expressed in the cortico-limbic pathways involved in the control of approach and avoidance behaviours [27]–[29]. Experimental manipulations of ECS elicit significant behavioural effects, especially in threat- and reward-related domains [30]. In particular, genetic deletion or inhibition of the enzyme fatty acid amide hydrolase (FAAH), the primary metabolic enzyme for AEA, produces context-dependent anxiolytic effects [31], [32] and a polymorphism of FAAH is linked to the divergent effects of threat-related stimuli on amygdala and of reward-related stimuli on striatum [2].

Given approach and avoidance are complex behaviours, they are probably determined by multiple factors, including neurochemical profiles, receptor expressions, and synaptic connection patterns. In particular, the present research was aimed at analysing the involvement of ECS on approach and avoidance behavioural processes. To this aim, we analysed the spontaneous behaviour of adolescent mice in an approach/avoidance conflict task related to seeking for a novel palatable food [10]. Adolescent animals are the most appropriate sample because they are reported to be statistically over-represented, when compared to adults, in the group showing a high responsivity towards conflicting situations [33]–[38]. In a previous study, we found evidence of enhanced or reduced CB_1_-mediated control on dorsal striatal GABAergic transmission associated with spontaneous approach/exploratory or avoidance behaviour, respectively, and inhibition of FAAH affected avoidance behaviour in the approach/avoidance conflict task [10]. Namely, the maximum reduction of the GABA_A_ receptor-mediated inhibitory postsynaptic currents by the CB_1_ receptor agonist HU210 in striatal slices was approximately 40%, 20% and 0% for the approaching (AP), balancing (BA) and avoiding (AV) animals, respectively. One explanation for these observations, particularly for the very large phenotypic effect upon GABA-ergic signalling is that the AP, BA and AV animals either express markedly different densities of CB1 receptors, and/or activities of FAAH in the brain regions involved in the behaviours. In consequence, we have investigated the density and functionality of CB_1_ receptors as well as FAAH activity in different brain regions in the three selected sub-populations of mice that exhibited avoidance or balanced or approach responses to conflicting stimuli in an approach/avoidance Y-maze.

## Results

### A/A Y-Maze in AV, BA and AP animals

**Figure 1:**
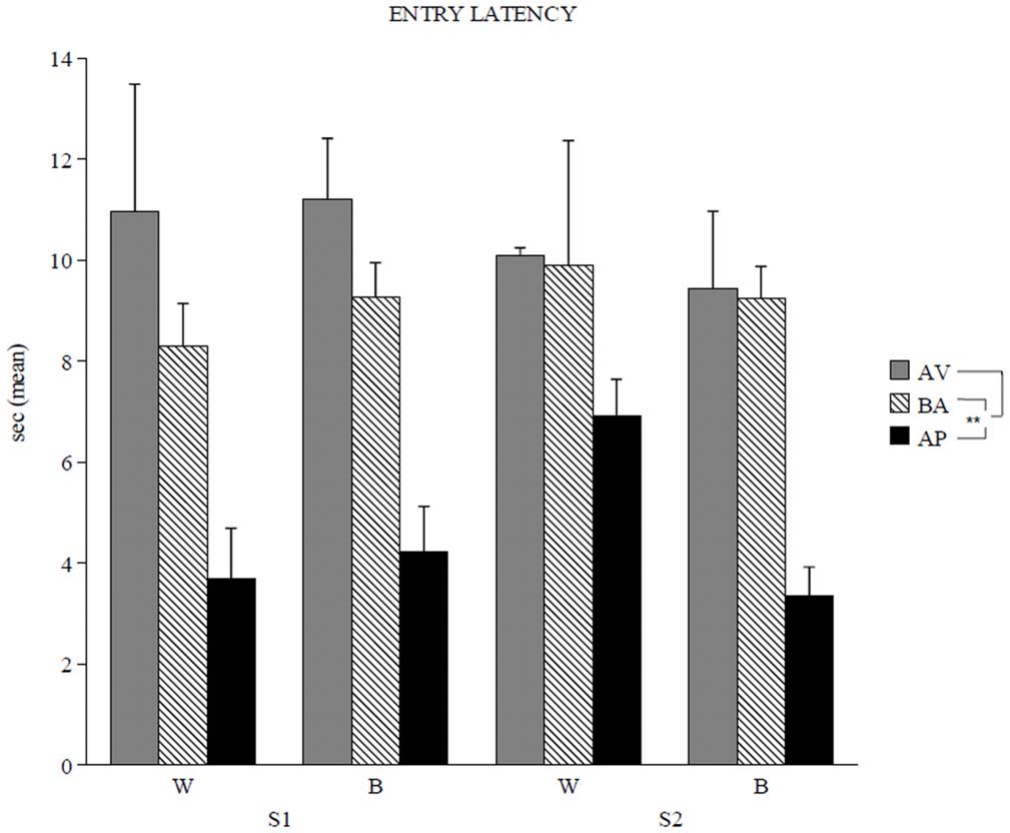
Entry latency of the avoiding (AV), balancing (BA) and approaching (AP) animals. High behaviour of approach exhibited by the AP animals was accompanied with impulsive/exploratory behaviour, as showed by their lower entry latency in comparison to BA and AV animals. Data are presented as means ± SEM. Asterisks indicate the significance level of the *post hoc* comparisons between AP vs. AV or BA groups: ** *P*<0.01. Abbreviations: white (W) and black (B) arms; first (S1) and second (S2) session.

When entry latency was analysed in relation to the categories the animals belonged to (Fig. 1), a three-way ANOVA (category x session x arm) revealed a significant category effect (*F*_2,12_ = 15.48, *P* = 0.0005), while session (*F*_1,12_ = 0.14, *P* = 0.71) and arm (*F*_1,12_ = 0.49, *P* = 0.49) effects were not significant. First- and second-order interactions were not significant. *Post hoc* comparisons on category effect revealed significant differences between AP and BA (*P* = 0.003) or AV (*P* = 0.0007) animals and no difference between AV and BA animals (*P* = 0.52). Thus, the behaviour of approach was accompanied with impulsive/exploratory behaviour.

### Autoradiography of [^3^H]CP55,940, CB_1_ receptor-mediated stimulation of [^35^S]GTPγS binding and FAAH activity in AV, BA and AP animals

**Figure 2:**
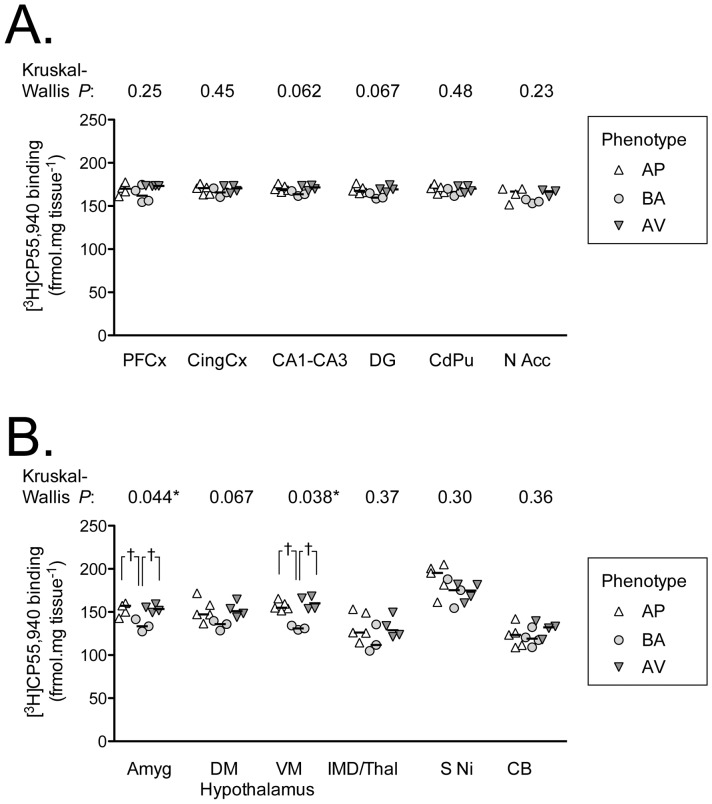
[^3^H]CP55,940 autoradiographic determination of CB_1_ receptor expression in avoiding (AV), balancing (BA), approaching (AP) animals. Panel A: prefrontal cortex (PFCx), cingulate cortex (CingCx), CA1-CA3, dentate gyrus (DG), caudate-putamen (CdPu), nucleus accumbens (N Acc). Panel B: amygdala (Amyg), dorso-medial (DM) and ventro-medial (VM) hypothalamus, intermediodorsal thalamic nucleus (IMD/Thal), substantia nigra (S Ni) and cerebellum (Cb). The *P* values shown above each graph are for non-parametric one-way ANOVAs for each region. †, *P*<0.05, Mann-Whitney Test U test following significant one-way ANOVAs.

Scatter plots of the densitometric values of [^3^H]CP55,940 binding are shown in Fig. 2. Kruskal-Wallis ANOVA analysis indicated significant differences among AV, BA and AP animals in centro-medial amygdaloidal nuclei (*H* = 6.26, *P* = 0.04) and in hypothalamic ventro-medial nucleus (*H* = 6.54, *P* = 0.038). When Mann-Whitney U Test was performed on the significant effects in amygdaloidal nuclei and hypothalamic ventro-medial nucleus, CB_1_ receptor density was increased in AV and AP animals in comparison to BA animals (amygdaloidal nuclei: AV *vs.* BA *z* = 2.12, *P* = 0.03; AP *vs.* BA *z* = −2.24, *P* = 0.03; AV *vs.* AP *z* = −0.24, *P* = 0.81; hypothalamic ventro-medial nucleus: AV *vs.* BA *z* = 2.12, *P* = 0.03; AP *vs.* BA *z* = −2.24, *P* = 0.03; AV *vs.* AP *z* = 0.73, *P* = 0.46), as reported in Fig. 2 B.

Importantly, in terms of the phenotypic differences seen in the striatum in electrophysiological experiments [10], there were no differences among the three groups of animals in the caudate-putamen. Also in the remaining brain areas no significant differences were found among the three groups of animals.

Because these results could have been influenced by the small sample size (although the variability of the data was rather low) we performed a bootstrap test to verify the reliability of the statistical differences. For each of the 13 areas analyzed, the empirical sampling distribution of the differences under the null-hypothesis was estimated through 10000 resamplings (with replacement). According to this empirical sampling distribution, only the significant differences in the CB1 receptor density found in amygdaloidal nuclei and in hypothalamic ventro-medial nucleus had a probability ≤0.05 of occurring by chance (amygdaloidal nuclei: AV *vs.* BA: *P* = 0.05; AP *vs.* BA: *P* = 0.05; AV *vs.* AP: *P* = 0.50; hypothalamic ventro-medial nucleus: AV *vs.* BA: *P* = 0.02; AP *vs.* BA: *P* = 0.03; AV *vs.* AP: *P* = 0.40). The results of the bootstrap analysis are shown in Table S1.

**Figure 3:**
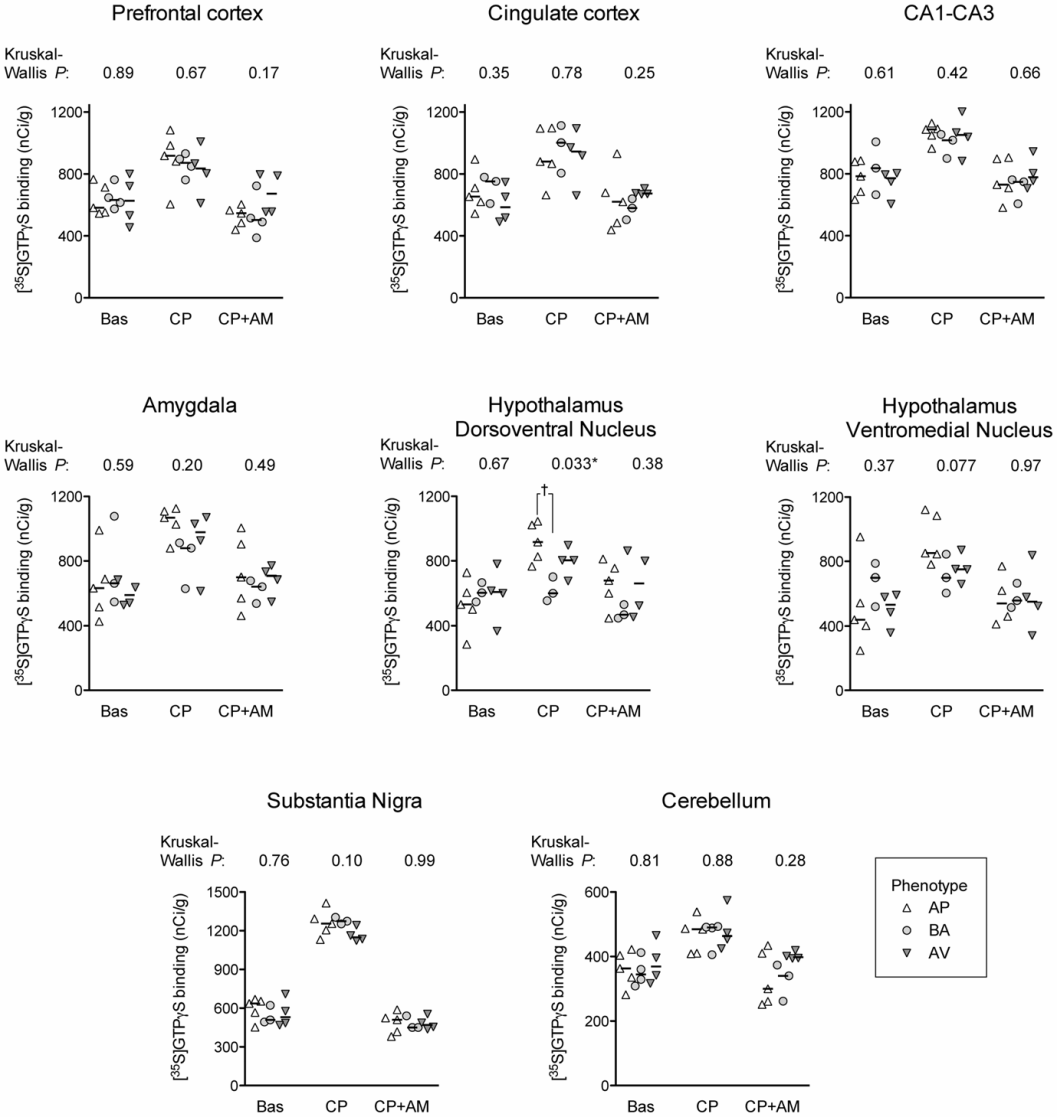
Basal (Bas), CP55,940- (CP) stimulated, and CP55,940+AM251 (CP+AM) treated [^35^S]GTPγS binding. The *P* values shown above each graph are for non-parametric one-way ANOVAs for each region. For the CP55,940-treated samples a significant effect was found in the hypothalamic dorso-medial nucleus. †, *P*<0.05, Mann-Whitney Test U test following significant one-way ANOVA. Abbreviations: avoiding (AV), balancing (BA) and approaching (AP) animals.

In the same regions, we measured CB_1_ receptor functionality, as assessed by agonist-stimulated [^35^S]GTPγS binding. Agonist-stimulated [^35^S]GTPγS binding can be expressed in many ways, such as % of control, increase over control, etc. We have elected to present the binding data as is, to allow the readers to see for themselves the magnitude of the increases in binding produced by the CB_1_ agonist used, and the loss of this increase when the agonist was co-incubated with the CB_1_ receptor-selective antagonist/inverse agonist AM251. Examples from eight brain regions are shown in Fig. 3. Kruskal-Wallis ANOVA performed on the basal or the CP55,940+AM251-treated [^35^S]GTPγS binding levels did not reveal differences among the three sub-populations of animals in any of tested brain regions. For the CP55,940-treated samples, however, a significant effect was found in the hypothalamic dorso-medial nucleus (*H* = 6.82, *P* = 0.03). In this area, AP animals showed higher CB_1_ agonist-stimulated [^35^S]GTPγS binding by 10 µM CP55,940 in comparison to BA animals (Mann-Whitney Test U: AP *vs.* BA: *z* = 2.24, *P* = 0.03; AP *vs.* AV: *z* = −1.47, *P* = 0.14; AV *vs.* BA: *z* = 1.77, *P* = 0.08), as reported in Fig. 3. Autoradiographic data for the caudate-putamen, nucleus accumbens, somatosensory cortex, and dentate gyrus were also analysed, but no significant differences among the three sub-populations of animals were found in these regions for either basal, CP55,940 stimulated or CP55,940+AM251 treated values (data not shown).

Because even these results could have been influenced by the small sample size, we performed again a bootstrap test on autoradiographic functional data, using the same bootstrap procedure. According to the empirical sampling distribution, the significant differences with a probability ≤0.05 of occurring by chance were found only for the CP55,940-treated samples of hypothalamic dorso-medial nucleus and also of the amygdaloidal nuclei (hypothalamic dorso-medial nucleus: AV *vs.* BA: *P* = 0.11; AP *vs.* BA: *P* = 0.03; AV *vs.* AP: *P* = 0.21; amygdaloidal nuclei: AV *vs.* BA: *P* = 0.27; AP *vs.* BA: *P* = 0.05; AV *vs.* AP: *P* = 0.18). The results of the bootstrap analysis are shown in Table S1.

**Figure 4:**
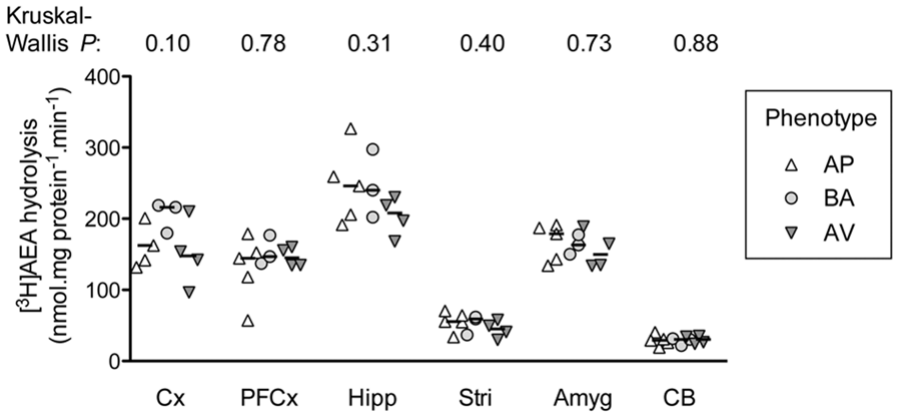
FAAH activities in avoiding (AV), balancing (BA) and approaching (AP) animals. FAAH assay values were not significantly different among AV, BA and AP mice in somato-sensory and motor cortex (Cx), prefrontal cortex (PFCx), hippocampus (Hipp), striatum (Stri), amygdala (Amyg) and cerebellum (Cb). The *P* values shown above the graph are for non-parametric one-way ANOVAs for each region.

FAAH activities were measured in six brain regions (cortex, parietal cortex, hippocampus, striatum, amygdala and cerebellum). Kruskal-Wallis ANOVA and bootstrap test performed on FAAH assay values failed to reveal any significant difference among AV, BA and AP mice (Fig. 4).

## Discussion

Approach behaviour is typically displayed in the context of moving towards a desired goal, while avoidance behaviour is displayed in the context of moving away from threatening or novel stimuli [1]. In the current research, in the presence of the same conflicting stimuli, while the BA animals reacted with balanced responses between approach and avoidance, the AV or AP animals exhibited inhibitory or advance responses towards one of the conflicting inputs, respectively. Individual differences in approach and avoidance behaviour might be modulated by the normal variance in the level of functioning of different systems, such as dopaminergic, serotoninergic, noradrenergic and endocannabinoid systems as well as many peptides, such as corticotropin releasing hormone [39], [40]. These substances act at various target areas to modulate neural transmission and to increase intensity of appetitive or defensive motivation [41]. Excessive activity of these systems has been related to psychopathological disorders, such as attention-deficit/hyperactivity disorders (ADHD), depression and anxiety [42]–[45].

The likelihood of a subject to be engaged in approach or avoidance motivation varies across situations, individuals and lifespan. One of the most striking example of the variability across lifespan is represented by the well-known observation that the adolescents show prominent motivation towards novel sensations, increased reward-responsivity and impulsivity as well as emotional lability and increased vulnerability to affective illness and addiction [33]–[37], [46]–[48]. Age-dependent differences in the brain levels of endocannabinoids as well as in the CB_1_-mediated effects on synaptic transmission have been described [49]–[51]. Notably, the characteristics linked to individual differences present in adolescence can become persistent traits maintained in adulthood [52], [53]. ECS plays a central role in the control of balancing action between approach and avoidance in both humans [5], [6] and rodents [7], [8], [10] and modulates GABAergic inhibition controlling fine-tuned behaviours [10], [54].

The present study was aimed at determining whether the different behavioural phenotypes showed markedly different CB_1_ receptor density and/or different activities of the AEA-metabolising enzyme FAAH in the brain. Since significant changes in receptor density do not necessarily translate into gross changes in receptor functionality, given the presence of receptor reserve, particularly in regions such as the hippocampus [55], [56], we have also investigated CB_1_ receptor functionality. The feasibility of the approach linking behaviour to biochemistry is supported by a recent study reporting differences in CB_1_ receptor density, CB_1_ receptor signalling (cannabinoid-induced ERK phosphorylation) and FAAH activity in relation to different reward sensitivities to palatable food [57].

At the outset, it should be pointed out that the group sizes were of necessity rather small as the approach and avoidance behaviours under study are extreme. The small sample size, however, was not unreasonable, since we were primarily interested in determining whether the robust differences in behaviour (and in the electrophysiological synaptic responses in dorso-striatal slices) [10] were matched by large differences in ECS signalling. Once controlled and checked stability of the autoradiographic results by means of bootstrap tests, we have presented them as scattergrams to allow the readers to make up their own minds about their reliability. We found in both AP and AV animals higher CB_1_ receptor density in the amygdaloidal nuclei and in the hypothalamic ventro-medial nucleus in comparison to BA animals. An intriguing parallel on the relation between opposite temperamental traits and similar receptor availability is provided by a recent study that by using PET to record [^11^C]raclopride binding showed that the dopamine D_2/3_ receptor striatal availability was lower in healthy subjects with high or low sensation-seeking in comparison to subjects with average sensation-seeking [58].

Furthermore, the AP animals displayed an increase of exploratory/impulsive behaviours, as indicated by low entry latencies in A/A Y-Maze test, as well as higher CB_1_ receptor functionality in amygdaloidal nuclei and hypothalamic dorso-medial nucleus. In contrast, the three sub-populations of animals show similar FAAH activity.

Animal and human studies indicate that the amygdala is involved in processing biologically significant stimuli, in conditioned fear learning [59], [60], in emotional memory [61], [62], in assessment of novelty [63], [64], ambiguity [65], threat [66] and danger stimuli [59], [66] as well as in mood and personality dimension variability [2]. Consistently, our results show that when the stimuli were evaluated as having high motivational salience, amygdaloidal CB_1_ receptor density was increased, as occurring in the case of AV and AP animals (but not in BA animals). In the amygdala, CB_1_ receptors presynaptically inhibit mainly GABAergic neurotransmission [23]. In theory, in both AV and AP animals a changed inhibitory neurotransmission due to a difference in CB_1_ receptor expression could influence the amygdaloidal outputs, which are critical in processing emotionally salient stimuli, whether they are pleasant or aversive [4], [67]. These outputs converge on other limbic regions, such as hypothalamus, which mediate the autonomic and somatic components of overt action. In particular, the ECS plays a critical role in the hypothalamic control of energy homeostasis [25] and in the regulation of ingestive behaviour [68]. Again in theory, the changed CB_1_ expression in the hypothalamic nuclei showed by both AV and AP animals could affect their complex autonomic and somatic responses. We are aware, however, that the different behavioral patterns might be accounted for by other possible changes. A differential tuning of the ECS might lead even to long-term interference in neuronal and synaptic plasticity and produce stable changes in the structure and function of various neuronal circuits, besides a modulation of the ECS itself alone. Such a possibility unfortunately can not be revealed by studies of CB_1_ density and functionality, or enzymatic activity of endocannabinoid metabolic systems.

In a very recent paper [10] we demonstrated that in AP animals increased hedonic response and impulsive behaviour were linked to increased CB_1_-mediated presynaptic control on dorso-striatal inhibitory neurotransmission. Conversely, in AV animals increased inhibitory response to reward and withdrawal behaviour were linked to decreased CB_1_-mediated presynaptic control on dorso-striatal inhibitory neurotransmission. The current study indicates that the robust differences in the striatal CB_1_ receptor-mediated postsynaptic currents in the different behavioural phenotypes are not a direct consequence of striatal CB_1_ receptor expression levels, but rather reflect more subtle changes in ECS signalling.

A final consideration has to be advanced. In the current research appetitive and aversive motivational responses are accompanied with the same increased CB_1_ receptor density in the amygdaloidal-hypothalamic motivational circuits. Avoidance behaviour may be considered a form of approach, “approach to safety” [69], and thus both motivational behaviours may involve similar brain regions and mechanisms [4]. Given that the motivational systems have evolved primarily to support the organisms' drives and to direct action, their outputs facilitate information intake, motor recruitment and action readiness, leading to the centrality of physiological expression in affective engagement. Thus, the motivational reactions, initiated from sub-cortical structures connected with a number of regions implicated in arousal (amygdala-hypothalamus) and action (amygdala-dorsal striatum) are the foundation of emotional experience. The nodes of this network are strongly interconnected and on these nodes endocannabinoid signalling could influence the final behavioural output, by modulating weights of the various nodes.

## Materials and Methods

### Subjects

Eighty male adolescent (32±2 pnd) C57BL/6JOlaHsd mice (Harlan, Italy) were used in the present study. The animals arrived to the animal house at CERC/Santa Lucia Foundation, Rome, Italy, after weaning (21^th^ pnd). After their arrival from shipment, an at least 5-day adjustment period to the new home condition was allowed. The animals were housed four per cage, with food (Mucedola 4RF21, Italy) and water *ad libitum*, under a 12-h light/dark cycle with light on at 07:00 hours, controlled temperature (22–23°C) and constant humidity (60±5%).

### Ethics Statement

All efforts were made to minimize animal suffering and to reduce their number, in accordance with the European Community Council Directive of 24 November 1986 (86/609/EEC) and approved by the Ethical Committee on animal experiments of Santa Lucia Foundation.

### Experimental Procedures

All animals in the present study (initially n = 80) were tested in the Approach/Avoidance Y-Maze (A/A Y-Maze) test described below. On the basis of distribution curve of their spontaneous behaviour in the A/A Y-Maze, we selected AV (n = 5), BA (n = 5) and AP (n = 5) animals. The remaining 65 animals were excluded by the present study. Two weeks later, the 15 animals were killed to perform studies on the ECS. In detail, autoradiographic binding in the coronal brain sections was performed to analyse prefrontal cortex, motor and somatosensory cortices, nucleus accumbens, caudate-putamen, cingulate cortex, hippocampus, amygdala, hypothalamus, thalamus, substantia nigra and cerebellum. Furthermore, the FAAH assays in homogenates of the prefrontal cortex, striatum, hippocampus, amygdala and cerebellum, were also performed.

Because some brain tissues were damaged by the freezing process used, we analysed density and functionality of CB_1_ receptors and FAAH activity in AV (n = 3 for nucleus accumbens; n = 4 for the remaining brain areas), BA (n = 4 for prefrontal cortex and cerebellum; n = 3 for the remaining brain areas), AP (n = 4 for nucleus accumbens; n = 5 for the remaining brain areas) animals.

### Behavioural Testing: Approach/Avoidance Y-Maze (A/A Y-Maze)

The apparatus consisted of a Plexiglas Y-maze with a starting gray arm from which two arms (8 cm wide, 30 cm long and 15 cm high) stemmed, arranged at an angle of 90° to each other. A T-guillotine door was placed at the end of the starting arm to prevent the animal moving backwards. An arm entry was defined as four legs entering one of the arms. The two arms of choice differed both in colour and brightness. In fact, one of the two arms had black and opaque floor and walls and no light inside, while other had white floor and walls and was lit by a 16-W neon lamp. Notably, the coloured “furniture” as well as the neon lamp were exchangeable between arms to alternate the spatial position of the white and black arms. The apparatus was placed in a slightly lit room by a red light (40 W) and it was always cleaned thoroughly with 70% ethanol and dried after each trial to remove scent cues. At the end of each arm of choice there was a blue chemically-inert tube cap (3 cm in diameter, 1 cm deep) used as food tray. The depth of the tray prevented mice from seeing the reward at a distance but allowed for an easy reward, i.e., eating as well as the appreciation of reward scent, not reducing the olfactory cues.

Since the appetites for palatable foods have to be learned [8], [70], a week before behavioural testing the animals were exposed to a novel palatable food (Fonzies, KP Snack Foods, Munchen, Germany) in their home cages for three consecutive days [71]. Fonzies (8% protein, 33% fat and 53% carbohydrate, for a caloric value of 541 kcal/100 gm) consisted of corn flour, hydrogenate vegetable fat, cheese powder and salt.

At the beginning of behavioural testing, mice were subjected to 1-day habituation phase in which all Y-Maze arms were opened to encourage maze exploration. During habituation, no food was present in the apparatus. At the end of this phase and during the successive testing phases, to increase the motivation to search for the reward, 12 h before exposure to the experimental set-up, the animals were slightly food deprived by limiting the food access to 12 hours/day. Such a regimen resulted in no significant body weight loss, as indicated by body weight measures performed at the end of habituation phase and before S1 and S2. Testing phase starting 24 h after the habituation phase at 8 a.m. consisted of two 10-trial sessions. In the Session 1 (S1), the animal was placed in the starting arm and could choose to enter one of the two arms, both containing the same standard food reward. After eating the animal was allowed to stand in its cage for 1 min-inter-trial interval. At the end of each trial the reward was always replaced. The spatial position of each arm (black and dark or white and lighted) was side balanced during the whole test, to exclude any side preference.

During the Session 2 (S2) starting 24 h after S1, the white arm was rewarded with the highly palatable food (Fonzies), while the black arm was rewarded with the standard food pellet. Notably, this approach/avoidance test required to choose between two conflicting drives, reaching a palatable reward placed in an aversive (white and lighted) environment or reaching a standard food placed in a reassuring (black and dark) environment. In particular, as previously reported [10], the A/A conflict index, considered as the difference in the number of white choices between S1 and S2, was normally distributed (mean = Δ+1, SD = ±1.67). Thus, we identified mice belonging to the three different behavioural categories: - BA animals; - AV animals; - AP animals by using A/A conflict index as a measure. In particular, BA animals (25% of the sample) reacted with balancing responses between approach and avoidance and their values in the A/A conflict index corresponded to distribution mean. The two opposite tails of the curve represented the few subjects exhibiting responses unbalanced toward one of the conflicting inputs: AV animals (6% of the sample) exhibiting avoiding responses had values in the A/A conflict index corresponding to minus two standard deviations of the mean, while AP animals (6% of the sample) exhibiting approaching responses showed values in the A/A conflict index corresponding to plus two standard deviations of the mean. To analyse explorative behaviour, entry latency in the arm of choice was also considered.

### [^3^H]CP55,940 and [^35^S]GTPγS autoradiography

Coronal brain sections (20 µm) were obtained from AV, BA and AP animals. [^3^H]CP55,940 binding autoradiography was performed by incubating brain sections with 3 nM [^3^H]CP55,940 and defining the non-specific binding with 10 µM WIN55212-2 (Tocris Bioscience, Ellisville, MO, USA) [72], [73]. [^35^S]GTPγS autoradiography was performed as previously described [74], [75] assessing the stimulation of [^35^S]GTPγS binding by incubating brain sections with 0.04 nM [^35^S]GTPγS under the following conditions: in the absence (basal binding) or in the presence of 10 µM of the CB_1_ agonist CP55,940 (Tocris Bioscience) (stimulated binding), in the presence of 10 µM CP55,940+10 µM AM251 (Tocris Bioscience) and in the presence of 10 µM GTPγS (non-specific binding). [^3^H]CP55,940 (174.6 Ci/mmol) and [^35^S]GTPγS (1250 Ci/mol) were purchased from Perkin Elmer (Waltham, MA, USA).

Autoradiographic densities were determined in a blinded manner by densitometry using the Scion Image software (Scion Corporation, Frederick, MD, USA). Relative optical density values of brain regions were averaged over two consecutive sections per animal (bilateral readings) and converted to nCi/mg of tissue ([^3^H]CP,55940 receptor binding) using [^3^H] microscale standards (American Radiolabeled Chemicals inc, St. Louis, Mo, USA). Bilateral relative optical density readings were taken from consecutive sections for the different experimental conditions and converted to nCi/g of tissue ([^35^S]GTPγS binding) using [^14^C] microscale standards (GE Healthcare Biosciences, Piscataway, NJ, USA). CB_1_ receptor autoradiographic densities are given in fmol/mg tissue equivalent (fmol/mg tissue) and CB_1_ agonist-stimulation of [^35^S]GTPγS binding data are presented as nCi/g tissue. Analysis of the [^3^H]CP,55940 and [^35^S]GTPγS binding data were performed using the computer program PRISM, version 5.0c for the Macintosh (GraphPad Software, San Diego, CA, USA).

### FAAH assay

Brain regions of interest were scraped from coronal brain sections (4 sections per brain region and animal) using G25 needles (BD Biosciences, Frankin Lakes, NJ, USA) and placed into Eppendorf tubes containing Tris-HCl 10 mM pH 9. The brain regions were homogenised and frozen at −80°C until used for assay. FAAH was assayed using [ethanolamine-1-^3^H]AEA (specific activity 2.22 TBq mmol^−1^, American Radiolabeled Chemicals, Inc., St. Louis, MO) diluted with non-radioactive AEA (Cayman Chemical Co., Ann Arbor, MI, USA) to give an assay concentration of 0.5 µM [76]. Assays were run in a blinded manner using an assay protein concentration of 0.3 µg per assay, and an incubation time of either 50 or 90 min at 37°C. The data presented here are for the 50 min assays, but the same pattern of results was seen using a 90 min assay.

### Statistical Analysis

Behavioural data were compared by means of parametric three-way ANOVA (category x session x arm), followed by Tukey's HSD test and the results are given as mean ± SEM. Because of the small number of AV, BA and AP animals, the autoradiography and FAAH data are shown in the graphs as scatter plots with medians given, and statistical analyses undertaken with non-parametric methods (Kruskal-Wallis ANOVA with Mann-Whitney U Test when appropriate). Since sample sizes were small, the reliability of the statistical differences was assessed through a bootstrap procedure [77], [78] using 10000 resamplings, with replacement, from the full set of data. The differences were considered significant at the *P*≤0.05 level.

## Supporting Information

Table S1 **Summary of** ***P*** **values from bootstrap analyses of the [^3^H]CP55,940 binding and CP55,940-stimulated [^35^S]GTPγS binding data.** *P* values≤0.05 are shown in bold typeface.
(DOC)
