# Supplementary material for: CB1 Receptor Autoradiographic Characterization of the Individual Differences in Approach and Avoidance Motivation
Source: PLoS One. 2012 Jul 27;7(7):e42111. doi: 10.1371/journal.pone.0042111 (PMC3407173; doi:10.1371/journal.pone.0042111)
Supplement: Table S1 — Summary of P values from bootstrap analyses of the [3H]CP55,940 binding and CP55,940-stimulated [35S]GTPγS binding data. P values≤0.05 are shown in bold typeface. (DOC) [file pone.0042111.s001.doc]

|  |  |  |  |  |  |  |
| --- | --- | --- | --- | --- | --- | --- |
|  | **[^3^H]CP55,940 binding** | | | **CP55,940-stimulated [^35^S]GTPγS binding** | | |
|  | **AVxBA** | **AVxAP** | **BAxAP** | **AVxBA** | **AVxAP** | **BAxAP** |
| **Region** | ***P*** | ***P*** | ***P*** | ***P*** | ***P*** | ***P*** |
|  |  |  |  |  |  |  |
| prefrontal cortex | 0.16 | 0.37 | 0.22 | 0.40 | 0.31 | 0.42 |
| motor-somatosensory cortices | 0.21 | 0.40 | 0.27 | 0.42 | 0.30 | 0.39 |
| cingulate cortex | 0.37 | 0.48 | 0.38 | 0.35 | 0.47 | 0.36 |
| CA1-CA3 | 0.31 | 0.48 | 0.31 | 0.36 | 0.45 | 0.32 |
| dentate gyrus | 0.26 | 0.48 | 0.26 | 0.23 | 0.40 | 0.30 |
| caudate-putamen | 0.38 | 0.50 | 0.38 | 0.22 | 0.25 | 0.42 |
| nucleus accumbens | 0.35 | 0.47 | 0.34 | 0.31 | 0.42 | 0.33 |
| amygdala | **0.05** | 0.50 | **0.05** | 0.27 | 0.18 | **0.05** |
| dorso-medial hypothalamus | 0.11 | 0.49 | 0.07 | 0.11 | 0.21 | **0.03** |
| ventro-medial hypothalamus | **0.02** | 0.40 | **0.03** | 0.40 | 0.11 | 0.09 |
| thalamic nucleus | 0.14 | 0.45 | 0.11 | 0.28 | 0.25 | 0.10 |
| substantia nigra | 0.48 | 0.10 | 0.12 | 0.27 | 0.27 | 0.11 |
| cerebellum | 0.31 | 0.26 | 0.47 | 0.45 | 0.46 | 0.48 |
|  |  |  |  |  |  |  |
